# Factors shaping the gut bacterial community assembly in two main Colombian malaria vectors

**DOI:** 10.1186/s40168-018-0528-y

**Published:** 2018-08-27

**Authors:** Priscila Bascuñán, Juan Pablo Niño-Garcia, Yadira Galeano-Castañeda, David Serre, Margarita M. Correa

**Affiliations:** 1 Grupo de Microbiología Molecular, Escuela de Microbiología, Universidad de Antioquia, Medellín, Colombia; 2 Escuela de Microbiología, Universidad de Antioquia, Medellín, Colombia; 3 Institute for Genome Sciences, University of Maryland School of Medicine, Baltimore, MD USA

**Keywords:** Mosquito gut, Microbiota, Bacterial communities, High-throughput sequencing, Microbial ecology, Malaria vectors, Colombia, *Anopheles nuneztovari*, *Anopheles darlingi*

## Abstract

**Background:**

The understanding of the roles of gut bacteria in the fitness and vectorial capacity of mosquitoes that transmit malaria, is improving; however, the factors shaping the composition and structure of such bacterial communities remain elusive. In this study, a high-throughput 16S rRNA gene sequencing was conducted to understand the effect of developmental stage, feeding status, species, and geography on the composition of the gut bacterial microbiota of two main Colombian malaria vectors, *Anopheles nuneztovari* and *Anopheles darlingi*.

**Results:**

The results revealed that mosquito developmental stage, followed by geographical location, are more important determinants of the gut bacterial composition than mosquito species or adult feeding status. Further, they showed that mosquito gut is a major filter for environmental bacteria colonization.

**Conclusions:**

The sampling design and analytical approach of this study allowed to untangle the influence of factors that are simultaneously shaping the microbiota composition of two Latin-American malaria vectors, essential aspect for the design of vector biocontrol strategies.

**Electronic supplementary material:**

The online version of this article (10.1186/s40168-018-0528-y) contains supplementary material, which is available to authorized users.

## Background

In the past decades, a number of studies have demonstrated that mosquitoes, like many other organisms, harbor a microbiota that plays critical roles in their biology [1, 2]. In an attempt to understand its functions, microbial communities have been removed from the gut of laboratory-reared mosquitoes via antibiotics, resulting in the alteration of several mosquito life traits such as larval development [3–5], fecundity [6, 7], blood digestion [7], adult longevity [6–8], and even an increase on malaria parasite proliferation rates [7–10] has been observed. Further, studies performed in field-collected specimens have confirmed that mosquitoes rely on their gut microbiota for their development [11] and have highlighted the importance of understanding natural variations occurring in their gut microbiota, which strongly determine the mosquito competence to transmit malaria parasites [12].

Changes in the composition of the mosquito gut microbiota have been frequently attributed to variations in geography [11–14], seasonal climatic patterns [13–15], mosquito species [13–16], nutritional status [17–19], and developmental stage [19–21], but in some cases, the studies draw contradictory conclusions. For example, it has been shown that two *Culex* species harbor distinct microbiota assemblies, although dominated by few bacterial taxa [22], but no major differences have been observed in the gut microbiota composition of two *Anopheles* species [13, 15, 23] or even among different mosquito genera [15, 23]. Ambiguous results could be owed to various factors such as differences in sequencing techniques and depth, the use of culture-dependent vs culture-independent methods, and laboratory-reared vs field-collected mosquitoes, with microbiota being more diverse in the later [12, 24]. What appears evident from all studies is that bacterial communities of the mosquito gut change according to several factors, and most likely, the interplay of these factors determines the mosquito gut assemblies in natural conditions. Thereby, the aim of this study was to analyze simultaneously the effect of four factors upon the gut bacterial communities of two Colombian field-collected malaria vectors, *Anopheles darlingi* and *Anopheles nuneztovari.* To our knowledge, this is the first study that examines the gut microbiota of these two main Latin American malaria vectors using a high-throughput sequencing approach.

## Results

### *Anopheles* species composition and abundances

A total of 239 *Anopheles* specimens were collected in two municipalities of two important malaria-endemic regions of Colombia: Istmina (IST), located in the Pacific coast (PAC), western Colombia and El Bagre (BAG) in the Urabá-Bajo Cauca-Alto Sinú (UCS), northwest Colombia. Collections consisted of 116 female adults, 26 larvae, and four water samples from the larval habitats in BAG and 87 female adults, 10 larvae, and 3 water samples from larval habitats in IST (Table 1). In BAG, *A. darlingi* (49.1%) was the most abundant species among adults, whereas *A. nuneztovari* represented 16.4%, and other species accounted for ~ 35%. In IST, most adult mosquitoes belonged to the species *A. nuneztovari* (57.5%), followed by *A. darlingi* (42.5%). Of larvae collected in BAG, only 11.6% were *A. nuneztovari* and 3.8% *A. darlingi*. In IST, *A. darlingi* larvae predominated (70%) over *A. nuneztovari* (30%).

**Table 1:** *Anopheles* species composition in two malaria endemic regions of Colombia

Department/municipality	*Anopheles* species	Number of samples collected (%)	Number of samples sequenced
Antioquia/El Bagre	Adults	116 (100)	33
	*A. darlingi*	57 (49.1)	11 BF, 10 NBF
	*A. triannulatus*	30 (25.9)	ns
	*A. nuneztovari*	19 (16.4)	3 BF, 9 NBF
	*A. albitarsis* s.l.	7 (6)	ns
	*Anopheles* spp.	3 (2.6)	ns
	Larvae	26 (100)	4
	*A. darlingi*	1 (3.8)	1
	*A. triannulatus*	22 (84.6)	ns
	*A. nuneztovari*	3 (11.6)	3
	Water from larval habitats	4 (100)	4
Chocó/Istmina	Adults	87 (100)	30
	*A. darlingi*	37 (42.5)	7 BF, 6 NBF
	*A. nuneztovari*	50 (57.5)	7 BF, 10 NBF
	Larvae	10 (100)	9
	*A. darlingi*	7 (70)	6
	*An nuneztovari*	3 (30)	3
	Water from larval habitats	3 (100)	3

*BF* blood-fed females, *NBF* non-blood-fed females, *ns* not sequenced

### Sequencing data output

Eighty-three good-quality samples were selected for sequencing, of which 28 were blood-engorged female adult mosquitoes (BF), 35 non-blood fed adult females (NBF), 13 fourth instar larvae (L4), and seven water samples from the larval collection sites (Table 1). A MiSeq Illumina sequencing generated a total of 15,909,048 bacterial 16S rRNA gene raw reads which were assembled using FLASH [25] and filtered according to quality settings, producing a total of 8,120,490 reads. After applying the SWARM clustering algorithm [26], 1,453,332 unique sequences were identified, grouped into 274,990 unique swarms, and assigned to 14,440 unique operational taxonomic units (OTUs).

### Bacterial communities vary according to sample types: water samples from larval habitats, larvae, or adult guts

**Fig. 1:**
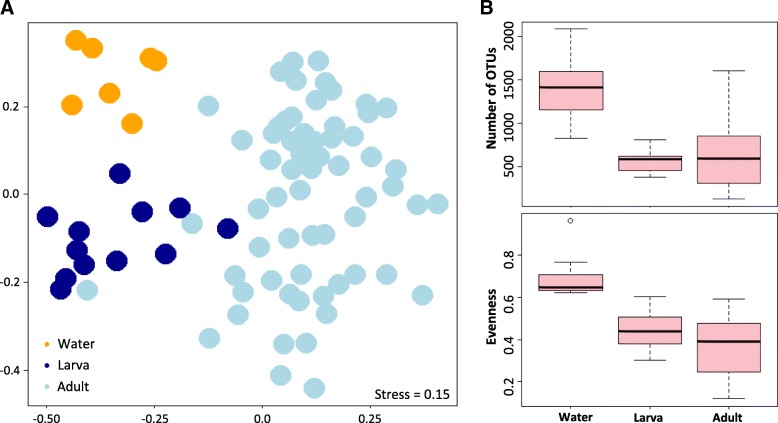
**a** Non-metric multidimensional scaling ordination—NMDS—showing that bacterial communities cluster by sample type using Bray-Curtis dissimilarity distance (see Shepard stress diagram in Additional file 1D). Each dot represents an individual collection of either a water sample, a larva, or an adult gut. **b** Boxplots representing the observed number of bacterial operational taxonomic units (OTUs) and OTUs evenness by sample type

Bacterial communities revealed differences across sample types, according to the collection environment: water samples (W), larvae guts (L), or adult guts (A) (Adonis, *R*^2^ = 0.16, *p* = 0.001). Interestingly, this clear segregation of bacterial communities according to the sample type (NMDS–ordination, Fig. 1a) was related with a sharp decline in the number of observed OTUs from the water samples (Mdn = 1417, IQR = 828–2081) to the larvae (Mdn = 584.5, IQR = 383–809) and adult specimens (Mdn = 594, IQR = 127–1605) (Fig. 1b). In addition, sample evenness followed the same declining pattern (Fig. 1b), as OTUs were less evenly distributed in larvae (Mdn = 0.40, IQR = 0.3–0.55) and adults (Mdn = 0.39, IQR = 0.12–0.59), than in the water samples (Mdn = 0.57, IQR = 0.46–0.72). In terms of the influence of geography, this factor showed a less pronounced effect (Adonis, *R*^2^ = 0.06, *p* = 0.001), with a larger overlap observed in the microbiome community composition (Additional file 1A). By contrast, no significant differences were detected in the gut bacterial community structures of the two *Anopheles* species (Additional file 1B, Adonis, *R*^2^ = 0.01, *p* = 0.14,) or the two adult feeding status (Additional file 1C, Adonis, *R*^2^ = 0.01, *p* = 0.44) analyzed. In addition, a Jaccard absence-presence-based analysis broadly supported these results (data not shown).

### Mosquitoes acquire most gut bacteria during the larval stage, but microbial composition varies across development

**Fig. 2:**
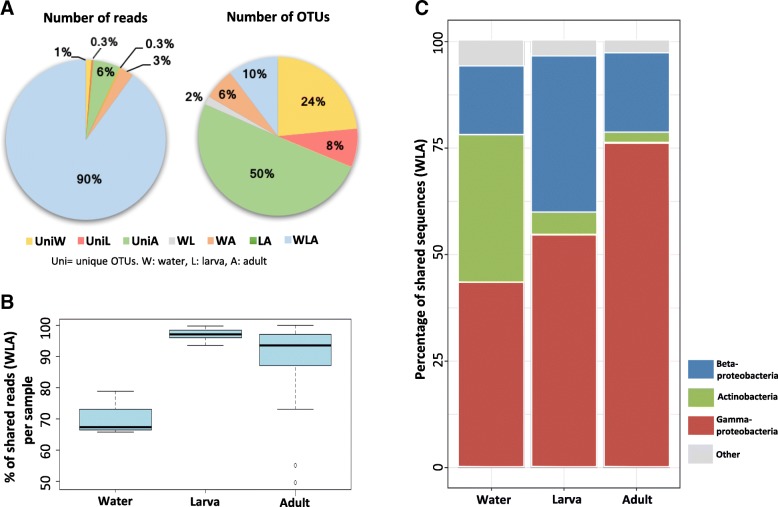
**a** Percentage of reads and OTUs that were unique or shared between sample types. OTUs shared by all groups accounted for 90% of the total reads and 10% of the total OTUs. **b** Boxplots showing the percentage of reads corresponding to OTUs shared among the three sample types (WLA). Not all shared OTUs are equally distributed across samples. **c** Relative abundances of the three most dominant bacterial classes: *Actinobacteria*, *Betaproteoabcteria* and *Gammaproteobacteria*, which vary across sample types

To elucidate the percentage of bacteria that are lost or gained across the lifecycle and those that can potentially persist transstadially, OTUs were subdivided according to their occurrence in one or more of the collection environments (i.e., sample types). The grouping was as follows: OTUs unique to water samples (uniW), larvae (UniL), or adults (UniA); OTUs shared among water samples and larvae (WL), water samples and adults (WA), larvae and adults (LA), and OTUs common to all sample types (WLA). This allowed to capture interesting patterns in the bacterial composition within and between sampled groups (Fig. 2a)*.* For instance*,* even when water samples were the least represented group (*n* = 7) and only constituted ~ 1% of the total number of reads, 24% of the total OTUs were unique to this group (UniW) (Fig. 2a). Similarly, 50% of the OTUs were exclusively present in adult guts (UniA); however, this percentage comprised < 6% of the total reads, in spite of the large number of samples (*n* = 62). Also, OTUs unique to larvae (UniL, *n* = 12) only accounted for the 8% of the total OTUs. Intriguingly, there was a lack of common OTUs between larvae and adult mosquitoes, which indicate that all OTUs common to these two groups were also present in the water samples. Finally, it was remarkable that 90% of the total reads were common to the three sample types (WLA); despite they only represented 10% of the total OTUs. For this reason, the subsequent analyses were focused on this bacterial subset.

Interestingly, the percentages of bacterial sequences within WLA group were not equally distributed across sample types, with 67.5% of reads per sample for the water samples and an approximate of 97% and 94% of reads per sample in larvae and adults, respectively (Fig. 2b). When looking at bacterial taxonomy, it was observed that the subset of shared bacteria (WLA) were represented by three main classes: *Actinobacteria*, *Betaproteobacteria*, and *Gammaproteobacteria*; however, strong variations in the relative abundances of these classes were detected across sample types (Fig. 2c). First, there was a gradual increase of the *Gammaproteobacteria* class in larval and adult mosquito guts with respect to the water samples. By contrast, there was a strong decline of *Actinobacteria* from the water samples to larval and adult mosquito guts. Finally, the relative abundance of the third class, *Betaproteobacteria*, showed its maximal occurrence in larval samples. These results indicate that although most of the aquatic bacterial colonizers persist transstadially from larvae to adults (WLA), the relative abundance of bacterial classes clearly change in the mosquito gut during the developmental stages.

**Fig. 3:**
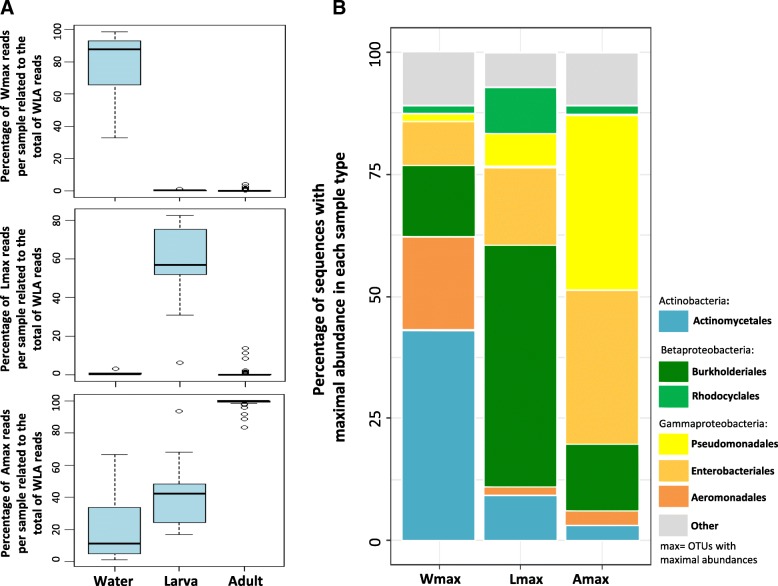
**a** Boxplots showing the percentage of shared OTU (WLA) reads per sample, with maximal abundances on each of the three sample types: water samples, larval guts, and adult guts. **b** Relative abundances of the most dominant OTUs with maximal abundance in each of the three sample types. OTUs were mainly represented by seven orders: *Actinomycetales*, *Burkholderiales*, *Rhodocyclales*, *Pseudomonadales*, *Enterobacteriales*, and *Aeromonadales*. Other OTUs represented ~ 10% of the total abundance

Further, OTUs categorized as WLA were present across sample types, but in most cases, their occurrences were low (most OTUs were present in only 1–20 out of 81 specimens; Additional file 2). However, when WLA OTUs were categorized according to the sample type where they showed a maximal abundance (Fig. 3), three clearly separated categories were found, which varied in their preferences for water sample (Wmax), larva (Lmax), or adult gut (Amax). In average, Wmax and Lmax OTUs consistently represented the highest proportion of the WLA reads in water samples and larvae (in average ~ 80% and ~ 60%, respectively) and had little representation in each of the other two sample types, respectively (Fig. 3a). Although Amax OTUs were coherently dominant in adults (i.e., more than 80% of WLA reads), they also represented up to 60% of the community in water samples and larvae.

Interestingly, distinctive bacterial assemblies were observed in the three groups analyzed (Wmax, Lmax, and Amax). Almost 50% of the bacterial relative abundance of the water samples corresponded to the order *Actynomicetales*, while this percentage strongly decreased in larvae (~ 9%) and adults (~ 2%). Moreover, a clear dominance of *Burkholderiales* for larval guts, with > 50% of relative abundance, and of *Pseudomonadales* and *Enterobacteriales* in adult guts was observed (Fig. 3b).

### Bacterial diversity of adult mosquitoes according to geography, *Anopheles* species or feeding status

**Fig. 4:**
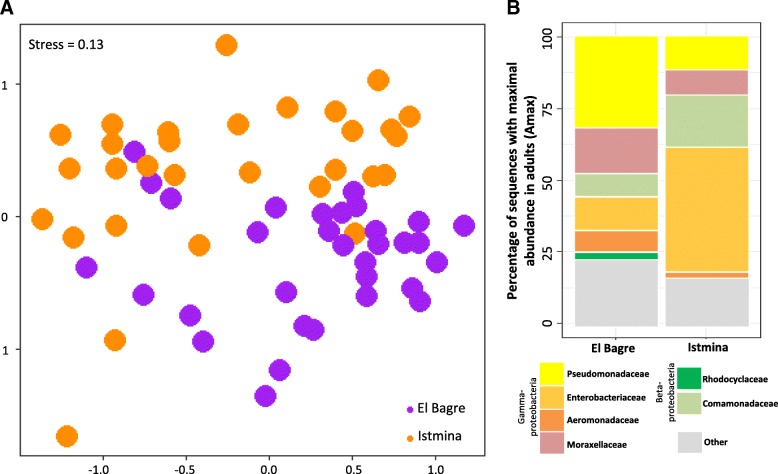
**a** Non-metric multidimensional scaling ordination showing how gut bacteria with maximal peak of abundance in adults, clustered by municipality (see Shepard stress diagram in Additional file 4C). **b** Taxonomical classification showing that adult mosquito guts are mainly populated by six families: *Enterobacteriacea*, *Comamonadaceae*, *Aeromonadaceae*, *Pseudomonadaceae*, *Moraxellaceaea*, and *Rhodocyclaceae*, with differences in their relative abundances

One of the main goals in studying the mosquito microbiome is to assess the variability of the bacterial community composition of the vectors of diseases such as malaria. In this study, a large dispersion on the bacterial community composition of the adults was observed (betadisper test, Additional file 3). To understand the possible factors responsible for the variation in the composition of Amax (identified as the largest pool of bacteria with clear preferences for *Anopheles* adults), the role of each of the following factors was analyzed: geography (Fig. 4), mosquito species (Additional file 4A), and female feeding status (Additional file 4B). Interestingly, results showed that bacterial communities significantly segregate into two main groups, one corresponding to mosquitoes collected in BAG and the other, to mosquitoes from IST (Adonis, *R*^2^ = 0.10, *p* = 0.001; Fig. 4a). Furthermore, classification into families showed that adults contained similar taxa, but their relative abundances differed (Fig. 4b). The six most abundant families comprised *Enterobacteriaceae*, *Comamonadaceae*, *Aeromonadaceae*, *Pseudomonadaceae*, *Moraxellaceae*, and *Rhodocyclaceae*; but of notice, guts of mosquitoes collected in BAG were largely inhabited by bacteria of the *Pseudomonadaceae* family (32%) and *Moraxellaceaea* (16%), whereas those from IST, by *Enterobacteriaceae* (~ 43%) and *Comamonadaceae* (~ 19%). In contrast, no clear differences were observed for the most abundant OTUs of the adult gut microbiota (Amax), when comparing between mosquito species (Adonis, *R*^2^ = 0.02, *p* = 0.26; Additional file 4A) or feeding status (Adonis, *R*^2^ = 0.01, *p* = 0.48; Additional file 4B). An additional non-abundance-based test was performed based on the Jaccard distance metric, which also showed a significant difference among localities, but not among species or feeding status (data not shown).

## Discussion

To collect food on water surfaces, the anopheline larva uses its head brushes to feed on bacteria, algae, protozoa, invertebrates, and detritus [27]. Mosquito larvae are usually not discriminatory in what they ingest; however, the size of particles is commonly < 50 μm [27]. Since bacteria are generally below 2 μm in diameter [28], most bacteria are potentially able to cross the mosquito brushes and enter the larval gut. Nevertheless, this study showed that only a fraction of the aquatic bacteria present in the water samples of the larval habitats was detected in the mosquito larval guts. This could be due to differences in sample coverage, but it might also suggest that a significant portion of the aquatic bacteria does not encounter larvae or is not able to survive inside the larval gut environment. Moreover, the results revealed that some OTUs entering the larval gut become dominant in this new habitat whereas a large proportion of them become rare or disappear, as shown by a concomitant decrease in richness and evenness from water samples to larvae (Fig. 1b). A previously proposed hypothesis states that the human gut environmental conditions select for a series of microbial traits that allow the survival and growth of certain environmental bacteria [29]. Following this idea, most aquatic bacteria could potentially be ingested by larvae, but a series of characteristics inherent to the mosquito gut would benefit the establishment of only a subset, depending on specific microbial traits. One possible selecting condition is the pH, since mosquito guts are mainly alkaline, in the range 8–11 [30, 31], due to high concentrations of carbonate ions [32], while in this study, the mean pH recorded at the waterbodies was 6.8 ± 0.3; signifying a drastic change for bacteria, from an acidic/neutral to a rather basic environment. Apart from pH, the redox potential and the presence of certain proteolytic enzymes and nutrients in the mosquito gut might be as well selective pressures upon early colonists [33]. Moreover, recent studies carried out in *Aedes* and *Anopheles* mosquitoes showed that the host genetics, in terms of immune and amino acid metabolic genes, is an important driver of the mosquito gut bacterial community structure [34, 35]. Deeper studies on the genetics and/or physiology of Latin American mosquitoes might help to elucidate the influence of these factors on the earliest mosquito gut microbial colonization. In addition, other ingested microorganisms or maternally transmitted bacteria may also compete with the new colonists and contribute to shaping the novel bacterial community. For instance, a mutual exclusion of the maternally inherited bacteria *Wolbachia* and the predominantly environmental bacteria *Asaia* has been described in the reproductive tract of the Asian malaria vector *Anopheles stephensi* [36]. Hence, the larval gut appears to act as a major filter for aquatic bacteria, by either limiting the establishment or enhancing the proliferation of some colonists. Interestingly, the taxonomical analysis in this study revealed that the most restricted bacterial taxa was *Actinobacteria*, which was the second most abundant class in the water samples and strongly declined in abundance in larvae and adult guts. *Actinobacteria* are mostly known as free-living microorganisms which, among others, play a critical role in the breakdown of plant biomass [37], role that could be carrying out in the waterbodies where larvae develop. It is also known that *Actinobacteria* can play different roles as symbionts of insect hots, although much less information is available. For instance, some *Actinobacteria* provide the Chagas vector *Rhodnius prolixus* with B-complex vitamins essential for its development [38]. Despite its low relative abundance in larvae, it could be hypothesized that the *Actinobacteria* present in the larval midgut might have a nutritional-associated role, for instance by contributing to the breakdown of ingested plant-based detritus present in the waterbodies where *Anopheles* larvae feed; however, *Beta* and *Gammaproteobacteria* were the two most successful groups in the larval gut environment, in particular, the order *Burkholderiales*.

Results from this and previous studies that have mainly analyzed African mosquitoes strongly indicate that bacteria acquired from the waterbody where mosquito larvae develop constitute the major gut bacterial community of adults [4, 12, 24, 39]. Also, a study conducted in field-collected *Culex* mosquitoes showed that more than 80% of the total sequences recovered were common to larval, pupal, and adult stages, indicating that most bacteria present in adults are transferred from larvae to adult [40]. In this work, close to 90% of the total reads obtained were common to water samples, larvae, and adult guts (WLA; Fig. 2a); however, the relative abundances of the taxonomic groups within the bacterial community varied from larvae to adults (Fig. 2c). A potential cause of such variation is that insect guts are unstable habitats due to the occurrence of several molts during development [41]. Particularly in mosquitoes, there is a complete metamorphosis process during which pupae dispose of one of two gut peritrophic matrixes, resulting in a significant gut microbial loss [42]. In fact, a culture-dependent study showed that the mean bacterial count of *Aedes triseriatus* midguts was reduced by approximately 280-fold between the larval and the pupal stages [43]. Also, variations on the gut bacterial community from larvae to adults may be a result of the drastic change on their diet. While larvae feed on aquatic detritus and microorganisms, female adult mosquitoes feed on nectar and vertebrate blood [44]. Based on results, it is likely that the mosquito metamorphosis, together with the diet shift from larvae to adults, represented a bottleneck for some taxa like *Betaproteobacteria*, which proliferated in larvae, but its relative abundance was reduced in adults, and signified a positive event for other bacteria like *Gammaproteobacteria*, which instead increased its abundance from larvae to adults (Fig. 2c). Overall, these changes imply that there is a rearrangement of the gut bacterial assembly during mosquito development.

Interestingly, for OTUs that were common to all sample types (WLA), their occurrence was low in most cases, suggesting that this subset of OTUs may have differential ecological preferences for each sample type (W, L, A). The comparison of the taxonomic composition between OTUs comprising Wmax, Lmax, and Amax categories revealed that half of the bacterial community of the water samples was composed of bacteria from the order *Actynomicetales*, while *Burkholderiales* clearly dominated the larval guts (Fig. 3b). Instead, adult guts were inhabited by similar proportions of *Pseudomonadales* and *Enterobacteriales*, both belonging to the *Gammaproteobacteria* class, and represented approximately 70% of the sequences of the group. In addition, approximately half of the total OTUs retrieved were found exclusively in adult gut samples (Fig. 2a); yet, several of these OTUs occurred in high abundances in a few adult specimens. This strongly suggests that the pool of bacteria present during adulthood is strongly related to the life history of each mosquito; for instance, adults acquire bacteria by their interaction with a variety of plant and animal sources during a nectar or a blood meal. Thus, even when most bacteria present even when most bacteria present in the adult gut are acquired in the early developmental stages from the aquatic reservoirs, many other bacterial taxa are also gained along the mosquito lifetime.

Understanding which bacteria are the most successful colonizers of adult guts, where potential interactions between bacteria and malaria parasites may occur, is essential for the design of biocontrol strategies. As shown in this study, bacteria with the highest abundances in adults (Amax) likewise represented a significant portion of the shared microbiota of larvae and water samples, suggesting that these bacteria might be suitable candidates to investigate as biocontrol agents. Nonetheless, in the search for potential biocontrol candidates, other factors should be considered, such as the effect of geography upon the mosquito gut microbiota. Results of this study showed a high variability in the bacterial microbiota of the adults and also that the adult mosquito guts were mainly inhabited by similar taxa at the family level; however, their relative abundances strongly varied across locations. The guts of adult mosquitoes from BAG were mainly inhabited by bacteria of the *Pseudomonadaceae* family, while those from IST were dominated by the *Enterobacteriaceae* family. Interestingly, field studies performed in the African and Asian vectors revealed that *Plasmodium*-infected and Chikungunya-infected mosquitoes, respectively, had higher abundances of *Enterobacteriaceae* than non-infected mosquitoes [12, 45]. Nonetheless, other studies have shown that some naturally isolated *Enterobacter* species severely affects *Plasmodium* development in African and American malaria vectors [14, 46, 47]. These apparent contradictory results might be explained by a possible intra-specific bacterial diversity. It has been shown for different isolates of the *Enterobacteriaceae Serratia marcescens*, which presented strong structural and phenotypic variations that directly correlated with the ability of this bacteria to inhibit the *Plasmodium* development within *Anopheles* mosquitoes [48]. What is remarkable in our results is that the mosquito guts of the IST municipality presented high levels of *Enterobacteriaceae* and that this municipality is located in Chocó, the department with the highest number of malaria cases in Colombia [49], rendering interest to perform further studies to understand whether a particular group of bacteria belonging to the *Enterobacteriaceae* family is influencing the *Plasmodium* survival. In contrast, little is known about the role of the *Pseudomonadacea* family, highly abundant in BAG mosquitoes, in the development of the malaria parasite. However, specific products extracted from different species of *Pseudomonas* have shown to reduce the longevity and fecundity of *A. stephensi* mosquitoes [50, 51].

Similar to this study, Akorli et al. [13] reported differences in the mosquito gut bacterial composition among localities but not among the African species *Anopheles gambiae* and *Anopheles coluzzii.* However, as mentioned above, other studies have shown differences across mosquito species and strains [4, 22, 34]. One of the characteristics of the present study is that the gut microbiota of two sympatric mosquito species, collected in two municipalities geographically separated, were simultaneously analyzed. This suggest that the genetic background that differentiates the two Colombian malaria vectors here analyzed, *A. darlingi* and *A. nuneztovari*, might not be a strong determinant of the gut bacterial microbiota composition.

Lastly, several studies have examined the influence of a blood meal upon mosquito bacterial communities, resulting in controversial conclusions. A culture-based study demonstrated that bacterial counts of the midgut of *Aedes triseriatus* mosquitoes increased by 70-fold 24 h after the blood meal, peaked 48 h after blood ingestion, and subsequently decreased 96 h after the blood meal [43]. Moreover, two high-throughput sequencing studies conducted in African *Anopheles* mosquitoes evaluated simultaneously the effect of collection site and female feeding status upon the microbiota composition, concluding that geographical location is a strong determinant of the bacterial composition, but reached opposite conclusions about the effect of blood meal on the gut microbiota of female adults [52, 53]. In the present study, in which other three factors were analyzed, the blood feeding status of female adult mosquitoes does not appear to be a strong determinant of the gut bacterial composition of the here analyzed mosquitoes; however, a more detailed analysis at the genus or species level could provide further information on the effect of this factor on the gut microbiota composition of malaria vectors.

## Conclusions

The design and approach of this study allowed the simultaneous evaluation of the interplay of different factors shaping the mosquito gut microbiota on field collected specimens. The results evidenced that (a) the mosquito gut is a major sieve for bacteria acquired from the aquatic environment; (b) mosquito gut bacteria are mostly acquired from the waterbodies where larvae develop; however, the mosquito developmental stage affects the structure of the gut bacterial community; and (c) the gut bacterial composition of adult mosquitoes is widely variable and seems to be associated to geography and mosquito life history, regardless of the species or blood feeding status. Finally, this study expanded the knowledge on the gut microbiota composition of two Latin American malaria vectors, *A. darlingi* and *A. nuneztovari*, through a next-generation sequencing approach.

## Methods

### Sample collection and mosquito identification

Samples were collected in two municipalities of two important epidemiological regions of Colombia (Additional file 5), during the months of January and September of 2015. The municipality of Istmina (5° 9′ N, 76° 41′ W) belongs to the Chocó Department and is situated in the Pacific Coast (PAC) region, western Colombia, while the municipality of El Bagre (7° 34′ N, 74° 48′ W) is part of the Antioquia Department and the Urabá-Bajo Cauca-Alto Sinú (UCS) region, northwest Colombia. Municipalities were selected because of their epidemiological importance, as Chocó is the first and Antioquia the third most affected departments in the country, both reported 65% (54,060) of the total malaria cases registered in 2016 [49]. Moreover, the species of interest, *A. nuneztovari* and *A. darlingi*, are the main malaria vectors in these areas with infection rates or percentage of *Plasmodium*-positive mosquitoes out of the total specimens collected, of < 1% in both areas and for both species [54–56]. Female adult *Anopheles* mosquitoes were collected from 18:00–24:00 h during three nights per locality, using 70% barrier screens [57] and human landing catches (HLC), under a protocol and informed consent approved by an Institutional Bioethics Committee of Sede de Investigación Universitaria-SIU, Universidad de Antioquia (reference number: 15-41-665). The use of both methods allowed collection of blood-fed and non-blood-fed mosquitoes. Fourth instar *Anopheles* larvae (L4) were collected from aquatic habitats using a dipper and were stored in water from the collection site until dissections. In addition, a 50 ml sample of the surface of waterbodies with presence of anopheline larvae was collected. Larvae and adult gut dissections were carried out under sterile conditions as follows: specimens were surface-sterilized in 70% ethanol for 2–3 min and rinsed three times in sterile 1× phosphate-buffered saline (PBS), which then served as sterility control. In addition, 70% ethanol tool cleansing was performed between sample dissections and an ethanol burner was placed close to the dissecting area. Guts were individually dissected and stored in 50 μl 70% ethanol and kept on ice or fridge until transfer to the lab settings. A posterior leg and the two wings of the adult specimens were mounted on a glass slide to aid species identification, using a morphological key [58]. An additional leg of each adult specimen and the rest of the body of larvae were individually preserved in 50 μl grinding buffer (10 mM Tris-HCl pH 8.2, 1 mM EDTA, 25 mM NaCl) for a rapid DNA extraction, posteriorly performed in the laboratory by adding Proteinase K (Mo Bio) to a final concentration of 200 μg/ml, incubating at 37 °C for 1 h and 95 °C for 5 min to inactivate the Proteinase K. DNA was used for molecular species confirmation by a polymerase chain reaction-restriction fragment length polymorphism—PCR-RFLP-ITS2 protocol [59, 60], with a 1:5 dilution of the larval DNA prior to PCR.

### DNA extraction and 16S rRNA gene amplification

DNA was extracted from gut or aquatic samples following a salt precipitation protocol [61]. For the Illumina library preparation, a two-step PCR amplification of the bacterial 16S rRNA gene hypervariable region 2 (V2) was carried out for each sample. The first amplicon was produced using primers Bact16S-101F (5′-AGYGGCGIACGGGTGAGTAA-3′) and Bact16S-338R (5′-TGCTGCCTCCCGTAGGAGT-3′), which comprised a 5′ oligonucleotide tail for the second PCR. The 50 μl reaction contained a final concentration of 1X Hi-Fi Reaction buffer, 1 mM dNTP mix, 0.4 μM of each primer, and 2 U of the high-fidelity velocity DNA polymerase (Bioline). The cycling conditions consisted of an initial denaturation at 98 °C for 30 s, followed by 30 cycles of denaturation at 98 °C for 10 s, annealing at 52 °C for 30 s, extension at 72 °C for 30 s with a final extension at 72 °C for 10 min. Amplicons were purified using the QIAquick 96 PCR purification kit (Qiagen), following manufacturer’s instructions. A second PCR was carried out to incorporate the Illumina adapter sequences and a six-nucleotide index (each index was distinct from all other indexes by at least two nucleotides) to each of the sequences using primers targeting the 5′ oligonucleotide tail; thus, each sample contained a unique barcode allowing directional sequencing (detailed in Additional file 6) The reaction contained a final concentration of 1× buffer, 1.25 U GoTaq (Promega), 2 mM MgCl2, and 2 μM of each primer under the following cycling conditions: initial denaturation of 94 °C for 3 min, followed by 15 cycles of 94 °C for 45 s, 50 °C for 45 s 72 °C for 45 s, and a final extension at 72 °C for 3 min. All indexed samples were subsequently pooled together, purified using the DNA Clean & Concentrator™-25 (Zymo Research), and sent to sequence. Purified water was used as negative control for all PCR reactions to monitor laboratory contamination. In addition, PBS remnants of field mosquito washings were used as sterility control of dissections and were processed and sequenced together with the rest of the samples.

### MiSeq sequencing and data analysis

A total of 83 samples consisting of 63 adult mosquitoes, 13 larvae, and 7 water samples from the larval habitats were paired-end sequenced by an Illumina sequencer server (Genome Quebec), using a MiSeq Reagent Kit v2 (500 cycles) with 25% PhiX content. Illumina paired-end reads were assembled with FLASH v1.2.11 [25] with a min/max overlap of 200/280 bp. Sequences were filtered according to the following quality parameters: a minimum 30 quality score over at least 80% paired sequence read and no ambiguous bases. Sequence clustering was performed to 81 good-quality samples through the robust method SWARM [26], and operational taxonomic units (OTUs) were assigned based on available sequences of the Ribosomal Database Project (RDP) [62, 63]. OTUs with < 5 reads were discarded to avoid spurious OTUs. Downstream analyses were performed in the R statistical software (version 3.4.0)[64], using the packages “Vegan” [65] and “ggplot2” [66]. First, chimera, archaea, mitochondria, and chloroplast reads were removed. Chimera were local reference-based detected with USEARCH [67, 68] and web-based classified using the RDP database. Then, as the number of reads varied among samples (mean ± SD = 82,144.96 ± 24,629.54), these were rarefied to a read depth of 39,983 reads per sample to homogenize the sampling effort. Nonetheless, given the ongoing debate regarding a possible effect of this type of normalization [69, 70], an exploratory analysis of the data, excluding the rarefying step, was also performed, with no strong differences detected in the patterns analyzed (see Additional file 7). Non-Metric Multidimensional Scaling (NMDS) analyses and ordination plots were performed to determine bacterial composition differences across sample types, localities, adult feeding status, and species, by calculating the Bray-Curtis dissimilarity index with 999 iterations. The function used (R: vegan: metaMDS) runs NMDS several times with random starting configurations, compares results, and stops after finding twice a similar minimum stress solution. In this function, the data are first square root transformed and then submitted to Wisconsin double standardization. Significant differences in the influence of each factor to the microbial composition across samples was determined using a multifactorial permutational multivariate analysis of variance (PERMANOVA, R: vegan: Adonis) [71], using both a Bray-Curtis and a Jaccard distance matrix with 999 permutations. The Shannon diversity index was calculated to obtain richness and evenness among sample types. Boxplots, pie charts, and stacked bar graphics were prepared to represent OTU richness, evenness, and relative abundance metrics. Specific scripts were designed to regroup OTUs in either unique or shared among sample types (Additional file 8), as well as to obtain the most abundant OTUs for each sample type (Additional file 9). These scripts were based on similar approaches that have been previously successfully applied in microbiome studies in a variety of environments [72–76].

## Additional files

Additional file 1: Non-metric multidimensional scaling 1. Non-metric multidimensional scaling ordinations of the microbiota composition of all OTUs with respect to geography **(A)**, species **(B)** and feeding status **(C)**. Shepard stress diagrams for all OTUs with respect to sample type and geography **(D)**, species **(E)** and feeding status **(F)**. (PDF 191 kb)

Additional file 2: Histogram. Histogram showing the frequency of shared OTUs (WLA) among samples. (PDF 171 kb)

Additional file 3: Betadisper output A) Boxplot of the distance to the centroid (i.e. dispersion) for each sample type. B) PCoA with polygons showing the dispersion in the bacterial community composition for different sample types. Notice the large dispersion in adults. (PDF 61 kb)

Additional file 4: Non-metric multidimensional scaling 2. Non-metric multidimensional scaling ordination of the most abundant bacterial OTUs, stacked bars representing the five most dominant bacterial families and table showing the 5 most abundant genera in adults (Amax), related to A) the mosquito species and B) the feeding status. C) Shepard stress diagram related to the NMDS of Amax sample subset. (PDF 239 kb)

Additional file 5: Map. Sampled municipalities belonging to two important epidemiological regions of Colombia: Urabá-Bajo Cauca-Alto Sinú (UCS) and the Pacific (PAC) regions. (PNG 169 kb)

Additional file 6: Barcoding primers. Detail of barcoding primers used for a directional sequencing. (DOCX 19 kb)

Additional file 7: Rarefied vs non-rarefied data. Non-metric multidimensional scaling ordinations, stacked bar plots and tables are shown as examples of the small variations observed after an exploratory analysis of the data comparing rarefied versus non-rarefied data. (PDF 241 kb)

Additional file 8: Script 1. Script designed to regroup OTUs in either unique or shared among sample types: water, larva and adult. (TXT 1 kb)

Additional file 9: Script 2. Script designed to obtain the most abundant OTUs for each sample type: water, larva and adult. (TXT 803 bytes)

## References

[1] Minard G, Mavingui P, Moro CV (2013). Diversity and function of bacterial microbiota in the mosquito holobiont. Parasit Vectors.

[2] Guégan M, Zouache K, Démichel C, Minard G, Tran Van V, Potier P (2018). The mosquito holobiont: fresh insight into mosquito-microbiota interactions. Microbiome.

[3] Mitraka E, Stathopoulos S, Siden-Kiamos I, Christophides GK, Louis C (2013). *Asaia* accelerates larval development of *Anopheles gambiae*. Pathog Glob Health.

[4] Coon KL, Vogel KJ, Brown MR, Strand MR (2014). Mosquitoes rely on their gut microbiota for development. Mol Ecol.

[5] Chouaia B, Rossi P, Epis S, Mosca M, Ricci I, Damiani C (2012). Delayed larval development in *Anopheles* mosquitoes deprived of *Asaia* bacterial symbionts. BMC Microbiol.

[6] Gendrin M, Rodgers FH, Yerbanga RS, Ouédraogo JB, Basáñez MG, Cohuet A (2015). Antibiotics in ingested human blood affect the mosquito microbiota and capacity to transmit malaria. Nat Commun.

[7] Sharma A, Dhayal D, Singh OP, Adak T, Bhatnagar RK (2013). Gut microbes influence fitness and malaria transmission potential of *Asian* malaria vector *Anopheles stephensi*. Acta Trop.

[8] Gendrin M, Yerbanga RS, Ouedraogo JB, Lefèvre T, Cohuet A, Christophides GK (2016). Differential effects of azithromycin, doxycycline, and cotrimoxazole in ingested blood on the vectorial capacity of malaria mosquitoes. Open Forum Infect Dis.

[9] Beier MS, Pumpuni CB, Beier JC, Davis JR (1994). Effects of para-aminobenzoic acid, insulin, and gentamicin on *Plasmodium falciparum* development in anopheline mosquitoes (Diptera: Culicidae). J Med Entomol.

[10] Dong Y, Manfredini F, Dimopoulos G (2009). Implication of the mosquito midgut microbiota in the defense against malaria parasites. PLoS Pathog.

[11] Coon KL, Brown MR, Strand MR (2016). Mosquitoes host communities of bacteria that are essential for development but vary greatly between local habitats. Mol Ecol.

[12] Boissière A, Tchioffo MT, Bachar D, Abate L, Marie A, Nsango SE (2012). Midgut microbiota of the malaria mosquito vector *Anopheles gambiae* and interactions with *Plasmodium falciparum* infection. PLoS Pathog.

[13] Akorli J, Gendrin M, Pels NAP, Yeboah-Manu D, Christophides GK, Wilson MD (2016). Seasonality and locality affect the diversity of *Anopheles gambiae* and *Anopheles coluzzii* midgut microbiota from Ghana. PLoS One.

[14] Tchioffo MT, Boissière A, Churcher TS, Abate L, Gimonneau G, Nsango SE (2013). Modulation of malaria infection in *Anopheles gambiae* mosquitoes exposed to natural midgut bacteria. PLoS One.

[15] Osei-Poku J, Mbogo CM, Palmer WJ, Jiggins FM (2012). Deep sequencing reveals extensive variation in the gut microbiota of wild mosquitoes from Kenya. Mol Ecol.

[16] Muturi EJ, Ramirez JL, Rooney AP, Kim CH (2017). Comparative analysis of gut microbiota of mosquito communities in central Illinois. PLoS Negl Trop Dis.

[17] Campbell CL, Mummey DL, Schmidtmann ET, Wilson WC (2004). Culture-independent analysis of midgut microbiota in the arbovirus vector *Culicoides sonorensis* (Diptera: Ceratopogonidae). J Med Entomol.

[18] Pumpuni CB, Demaio J, Kent M, Davis JR, Beier JC (1996). Bacterial population dynamics in three anopheline species: the impact on *Plasmodium* sporogonic development. Am J Trop Med Hyg.

[19] Wang Y, Gilbreath TM, Kukutla P, Yan G, Xu J (2011). Dynamic gut microbiome across life history of the malaria mosquito *Anopheles gambiae* in Kenya. PLoS One.

[20] Gimonneau G, Tchioffo MT, Abate L, Boissière A, Awono-Ambéné PH, Nsango SE (2014). Composition of *Anopheles coluzzii* and *Anopheles gambiae* microbiota from larval to adult stages. Infect Genet Evol.

[21] Kim CH, Lampman RL, Muturi EJ (2015). Bacterial communities and midgut microbiota associated with mosquito populations from waste tires in East-Central Illinois. J Med Entomol.

[22] Muturi EJ, Kim CH, Bara J, Bach EM, Siddappaji MH (2016). *Culex pipiens* and *Culex restuans* mosquitoes harbor distinct microbiota dominated by few bacterial taxa. Parasit Vectors.

[23] Andrews ES, Xu G, Rich SM (2014). Microbial communities within field-collected *Culiseta melanura* and *Coquillettidia perturbans*. Med Vet Entomol.

[24] Rani A, Sharma A, Rajagopal R, Adak T, Bhatnagar RK (2009). Bacterial diversity analysis of larvae and adult midgut microflora using culture-dependent and culture-independent methods in lab-reared and field-collected *Anopheles stephensi*-an Asian malarial vector. BMC Microbiol.

[25] Magoč T, Salzberg SL (2011). FLASH: fast length adjustment of short reads to improve genome assemblies. Bioinformatics.

[26] Mahé F, Rognes T, Quince C, de Vargas C, Dunthorn M (2014). Swarm: robust and fast clustering method for amplicon-based studies. PeerJ.

[27] Becker N (2010). Mosquitoes and their control.

[28] Lodish HF (2004). Molecular cell biology.

[29] Costello EK, Stagaman K, Dethlefsen L, Bohannan BJ, Relman DA (2012). The application of ecological theory toward an understanding of the human microbiome. Science (80-).

[30] del Pilar CM, VanEkeris L, Salazar MI, Bowers D, Fiedler MM, Silverman D (2005). Carbonic anhydrase in the adult mosquito midgut. J Exp Biol.

[31] Dadd RH (1975). Alkalinity within the midgut of mosquito larvae with alkaline-active digestive enzymes. J Insect Physiol.

[32] del Pilar CM, Fiedler MM, VanEkeris L, Tu C, Silverman DN, Linser PJ (2004). Alkalization of larval mosquito midgut and the role of carbonic anhydrase in different species of mosquitoes. Comp Biochem Physiol C Toxicol Pharmacol.

[33] Dillon RJ, Dillon VM (2004). The gut bacteria of insects: nonpathogenic interactions. Annu Rev. Entomol.

[34] Short SM, Mongodin EF, MacLeod HJ, Talyuli OAC, Dimopoulos G (2017). Amino acid metabolic signaling influences *Aedes aegypti* midgut microbiome variability. PLoS Negl Trop Dis.

[35] Stathopoulos S, Neafsey DE, Lawniczak MK, Muskavitch MA, Christophides GK (2014). Genetic dissection of *Anopheles gambiae* gut epithelial responses to *Serratia marcescens*. PLoS Pathog.

[36] Rossi P, Ricci I, Cappelli A, Damiani C, Ulissi U, Mancini MV (2015). Mutual exclusion of *Asaia* and *Wolbachia* in the reproductive organs of mosquito vectors. Parasit Vectors.

[37] Lewin GR, Carlos C, Chevrette MG, Horn HA, McDonald BR, Stankey RJ (2017). Evolution and ecology of Actinobacteria and their bioenergy applications. Annu Rev Microbiol.

[38] Baines S (1956). The role of the symbiotic bacteria in the nutrition of *Rhodnius prolixus* (Hemiptera). J Exp Biol.

[39] Dada N, Jumas-Bilak E, Manguin S, Seidu R, Stenström TA, Overgaard HJ (2014). Comparative assessment of the bacterial communities associated with *Aedes aegypti* larvae and water from domestic water storage containers. Parasit Vectors.

[40] Duguma D, Hall MW, Rugman-Jones P, Stouthamer R, Terenius O, Neufeld JD (2015). Developmental succession of the microbiome of *Culex* mosquitoes. BMC Microbiol.

[41] Engel P, Moran NA (2013). The gut microbiota of insects - diversity in structure and function. FEMS Microbiol Rev.

[42] Moll RM, Romoser WS, Modrzakowski MC, Moncayo AC, Lerdthusnee K (2001). Meconial peritrophic membranes and the fate of midgut bacteria during mosquito (Diptera: Culicidae) metamorphosis. J Med Entomol.

[43] Demaio J, Pumpuni CB, Kent M, Beier JC (1996). The midgut bacterial flora of wild *Aedes triseriatus*, *Culex pipiens*, and *Psorophora columbiae* mosquitoes. Am J Trop Med Hyg.

[44] Clements AN. The biology of mosquitoes: development, nutrition, and reproduction. London: Chapman & Hall; 1992.

[45] Zouache K, Michelland RJ, Failloux AB, Grundmann GL, Mavingui P (2012). Chikungunya virus impacts the diversity of symbiotic bacteria in mosquito vector. Mol Ecol.

[46] Cirimotich CM, Dong Y, Clayton AM, Sandiford SL, Souza-Neto JA, Mulenga M (2011). Natural microbe-mediated refractoriness to *Plasmodium* infection in *Anopheles gambiae*. Science (80-).

[47] Gonzalez-Ceron L, Santillan F, Rodriguez MH, Mendez D, Hernandez-Avila JE (2003). Bacteria in midguts of field-collected *Anopheles albimanus* block *Plasmodium vivax* sporogonic development. J Med Entomol.

[48] Bando H, Okado K, Guelbeogo WM, Badolo A, Aonuma H, Nelson B (2013). Intra-specific diversity of *Serratia marcescens* in *Anopheles* mosquito midgut defines *Plasmodium* transmission capacity. Sci Rep.

[49] Instituto Nacional de Salud (INS): Boletín epidemiológico semana 52.2017 November. http://www.ins.gov.co/buscador-eventos/BoletinEpidemiologico/2017%20Bolet%C3%ADn%20epidemiol%C3%B3gico%20semana%2052.pdf

[50] Parthipan P, Sarankumar RK, Jaganathan A, Amuthavalli P, Babujanarthanam R, Rahman PKSM (2017). Biosurfactants produced by *Bacillus subtilis* A1 and *Pseudomonas stutzeri* NA3 reduce longevity and fecundity of *Anopheles stephensi* and show high toxicity against young instars. Environ Sci Pollut Res Int.

[51] Prabakaran G, Hoti SL, Rao HS, Vijjapu S (2015). Di-rhamnolipid is a mosquito pupicidal metabolite from *Pseudomonas fluorescens* (VCRC B426). Acta Trop.

[52] Buck M, Nilsson LKJ, Brunius C, Dabiré RK, Hopkins R, Terenius O (2016). Bacterial associations reveal spatial population dynamics in *Anopheles gambiae* mosquitoes. Sci Rep.

[53] Tchioffo MT, Boissière A, Abate L, Nsango SE, Bayibéki AN, Awono-Ambéné PH (2016). Dynamics of Bacterial community composition in the malaria mosquito’s epithelia. Front Microbiol.

[54] Naranjo-Díaz N, Altamiranda M, Luckhart S, Conn JE, Correa MM (2014). Malaria vectors in ecologically heterogeneous localities of the Colombian Pacific region. PLoS One.

[55] Naranjo-Diaz N, Rosero DA, Rua-Uribe G, Luckhart S, Correa MM (2013). Abundance, behavior and entomological inoculation rates of anthropophilic anophelines from a primary Colombian malaria endemic area. Parasites and Vectors.

[56] Gutiérrez LA, González JJ, Gómez GF, Castro MI, Rosero DA, Luckhart S (2009). Species composition and natural infectivity of anthropophilic *Anopheles* (Diptera: Culicidae) in the states of Córdoba and Antioquia, Northwestern Colombia. Mem Inst Oswaldo Cruz.

[57] Burkot TR, Russell TL, Reimer LJ, Bugoro H, Beebe NW, Cooper RD (2013). Barrier screens: a method to sample blood-fed and host-seeking exophilic mosquitoes. Malar J.

[58] Gonzalez R, Carrejo N. Introducción al estudio taxonómico de *Anopheles* de Colombia: claves y notas de distribución. Cali: Programa Editorial Universidad del Valle; 2009.

[59] Cienfuegos AV, Rosero DA, Naranjo N, Luckhart S, Conn JE, Correa MM (2011). Evaluation of a PCR-RFLP-ITS2 assay for discrimination of *Anopheles* species in northern and western Colombia. Acta Trop.

[60] Zapata MA, Cienfuegos AV, Quirós OI, Quiñones ML, Luckhart S, Correa MM (2007). Discrimination of seven *Anopheles* species from San Pedro de Uraba, Antioquia, Colombia, by polymerase chain reaction-restriction fragment length polymorphism analysis of its sequences. Am J Trop Med Hyg.

[61] Rosero DA, Gutiérrez LA, Cienfuegos AV, Jaramillo LM, Correa MM (2010). Optimizacion de un procedimiento de extraccion de ADN para mosquitos anofelinos. Rev Colomb Entomol.

[62] Wang Q, Garrity GM, Tiedje JM, Cole JR (2007). Naive Bayesian Classifier for Rapid Assignment of rRNA Sequences into the New Bacterial Taxonomy. Appl Environ Microbiol.

[63] Cole JR, Wang Q, Fish JA, Chai B, McGarrell DM, Sun Y (2014). Ribosomal Database Project: data and tools for high throughput rRNA analysis. Nucleic Acids Res.

[64] R Core Team (2013). R: A language and environment for statistical computing.

[65] Oksanen J, Blanchet FG, Friendly M, Kindt R, Legendre P, McGlinn D (2017). vegan: Community Ecology Package.

[66] Wickham H (2009). ggplot2: elegant graphics for data analysis.

[67] Edgar RC (2010). Search and clustering orders of magnitude faster than BLAST. Bioinformatics.

[68] Edgar RC, Haas BJ, Clemente JC, Quince C, Knight R (2011). UCHIME improves sensitivity and speed of chimera detection. Bioinformatics.

[69] McMurdie PJ, Holmes S (2014). Waste not, want not: why rarefying microbiome data is inadmissible. PLoS Comput Biol.

[70] Weiss S, Xu ZZ, Peddada S, Amir A, Bittinger K, Gonzalez A (2017). Normalization and microbial differential abundance strategies depend upon data characteristics. Microbiome.

[71] Anderson MJM (2001). A new method for non-parametric multivariate analysis of variance. Austral Ecol.

[72] Shade A, Gilbert JA (2015). Temporal patterns of rarity provide a more complete view of microbial diversity. Trends Microbiol.

[73] Shade A, Jones SE, Caporaso JG, Handelsman J, Knight R, Fierer N (2014). Conditionally rare taxa disproportionately contribute to temporal changes in microbial diversity. MBio.

[74] Shade A, Handelsman J (2012). Beyond the Venn diagram: the hunt for a core microbiome. Environ Microbiol.

[75] Niño-García JP, Ruiz-González C, del Giorgio PA (2016). Landscape-scale spatial abundance distributions discriminate core from random components of boreal lake bacterioplankton. Ecol Lett.

[76] Saunders AM, Albertsen M, Vollertsen J, Nielsen PH (2016). The activated sludge ecosystem contains a core community of abundant organisms. ISME J.

