# Supplementary figures and images for: Factors shaping the gut bacterial community assembly in two main Colombian malaria vectors
Source: Microbiome. 2018 Aug 27;6:148. doi: 10.1186/s40168-018-0528-y (PMC6112144; doi:10.1186/s40168-018-0528-y)

**A**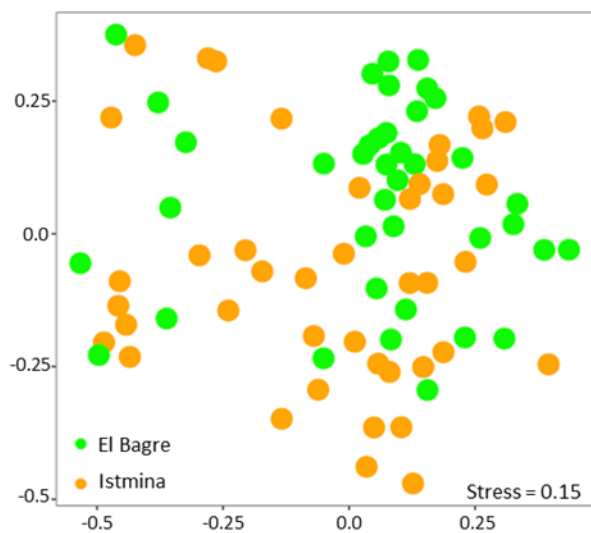**D**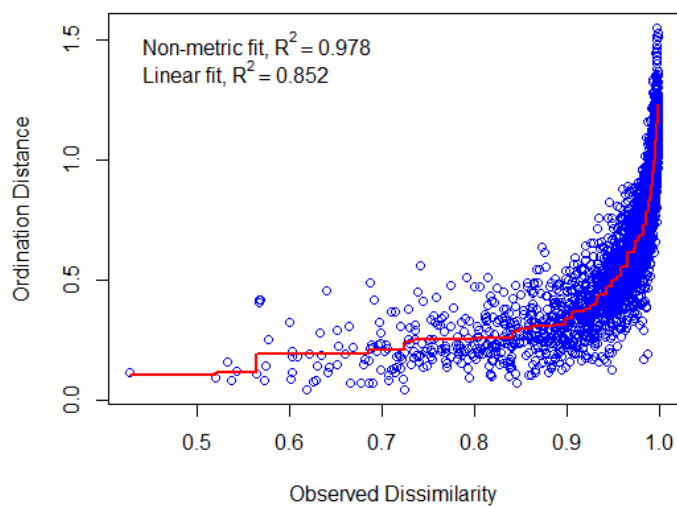**B**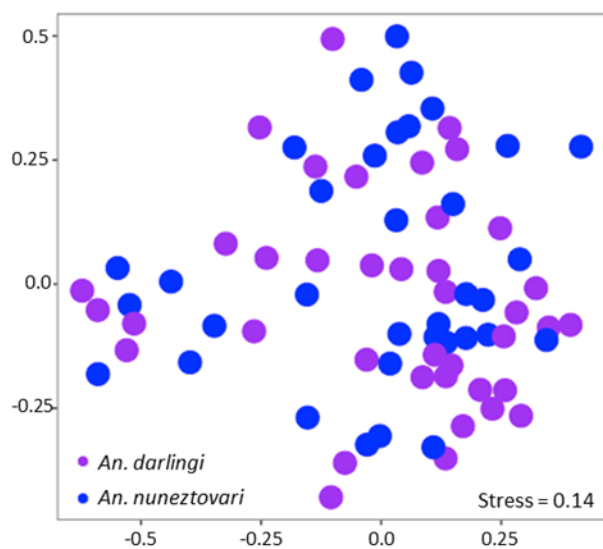**E**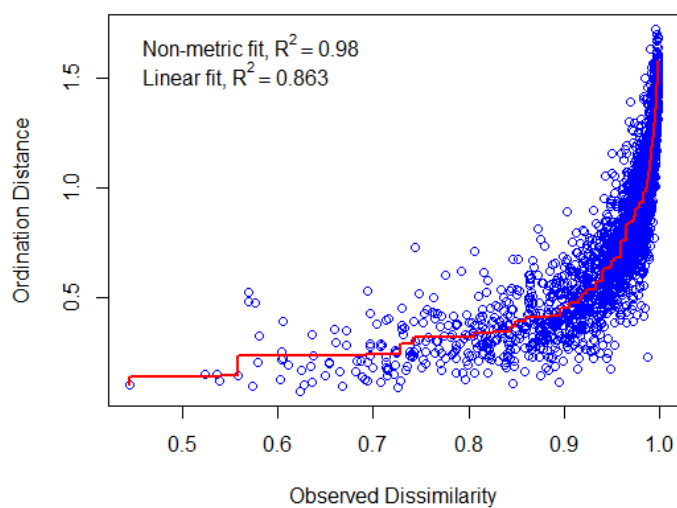**C**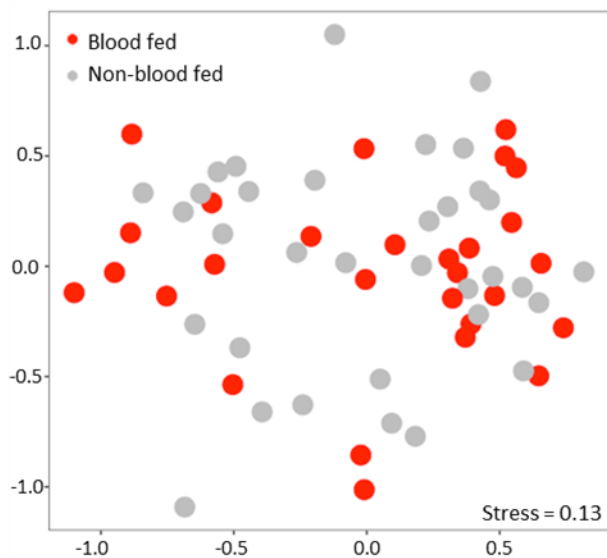**F**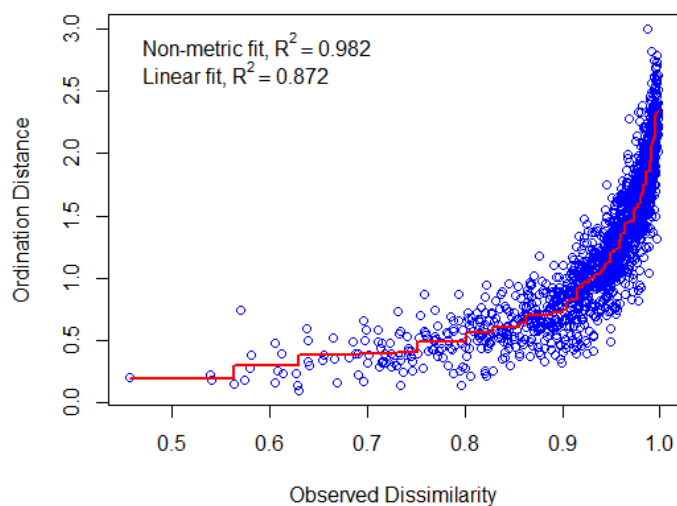

Supplement: Supplementary file 1 — Non-metric multidimensional scaling 1. Non-metric multidimensional scaling ordinations of the microbiota composition of all OTUs with respect to geography (A), species (B) and feeding status (C). Shepard stress diagrams for all OTUs with respect to sample type and geography (D), species (E) and feeding status (F). (PDF 191 kb) [file 40168_2018_528_MOESM1_ESM.pdf]

Frequency OTUs shared among sample types

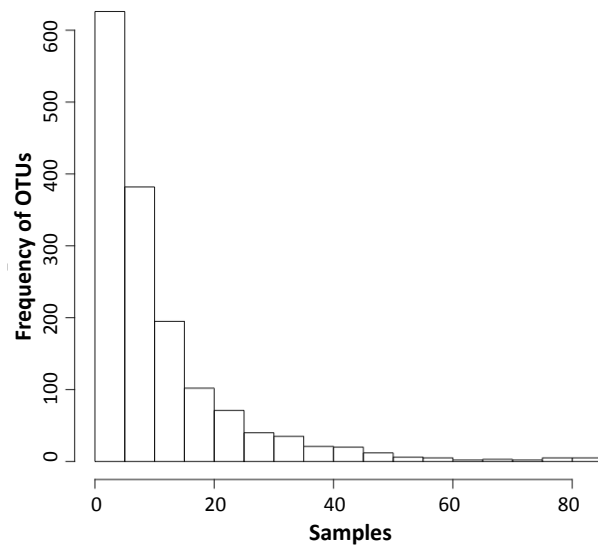

Supplement: Supplementary file 2 — Histogram. Histogram showing the frequency of shared OTUs (WLA) among samples. (PDF 171 kb) [file 40168_2018_528_MOESM2_ESM.pdf]

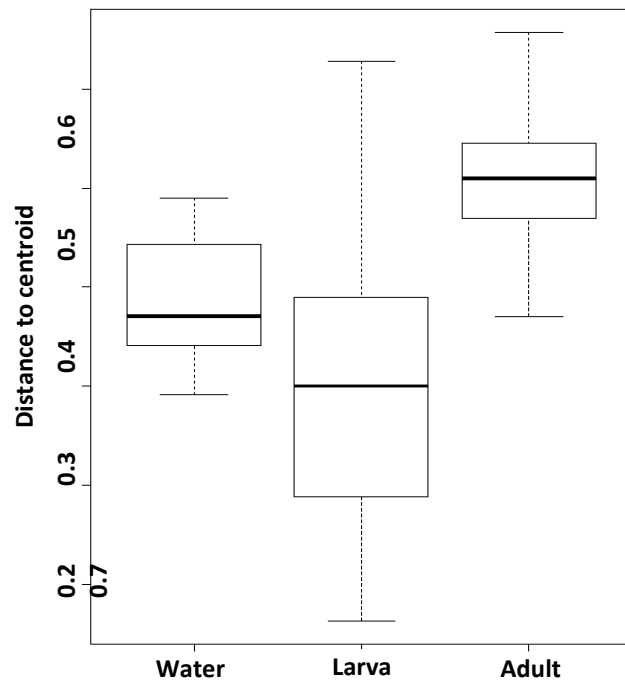

Supplement: Supplementary file 3 — Betadisper output A) Boxplot of the distance to the centroid (i.e. dispersion) for each sample type. B) PCoA with polygons showing the dispersion in the bacterial community composition for different sample types. Notice the large dispersion in adults. (PDF 61 kb) [file 40168_2018_528_MOESM3_ESM.pdf]

A

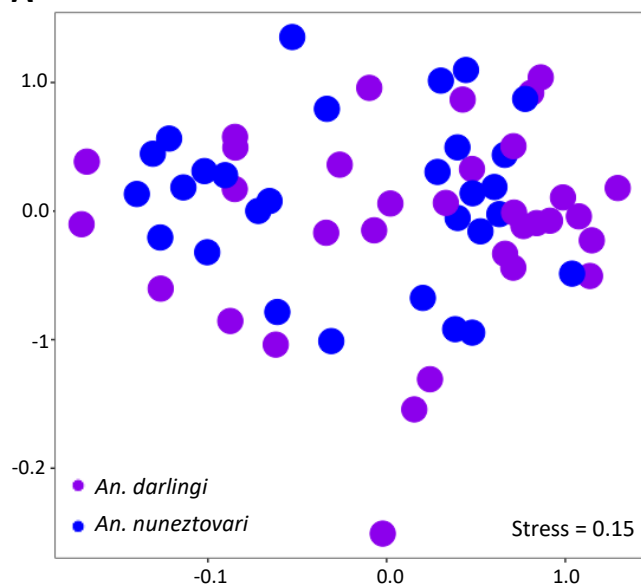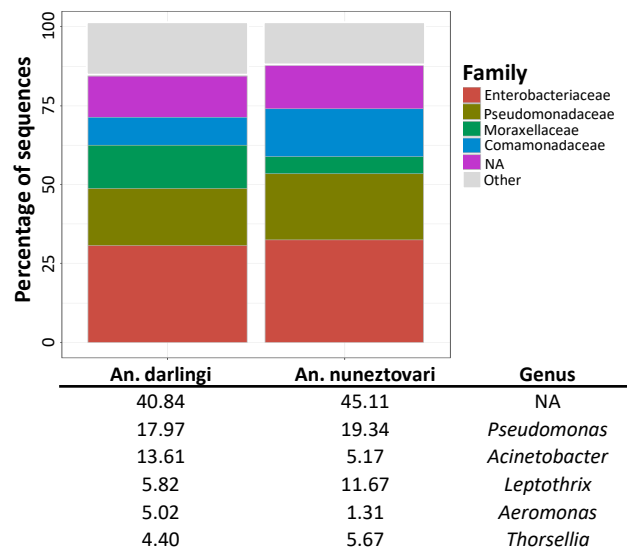

B

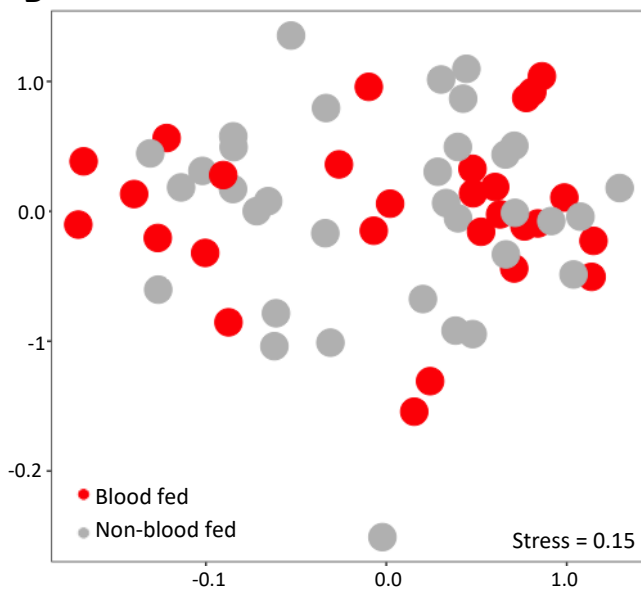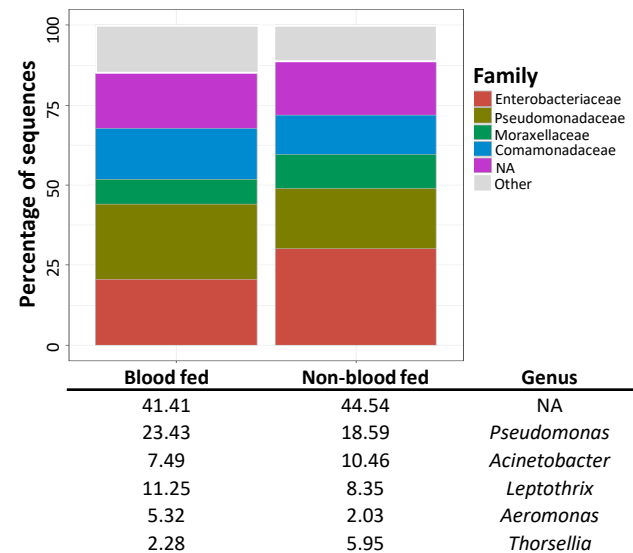

C

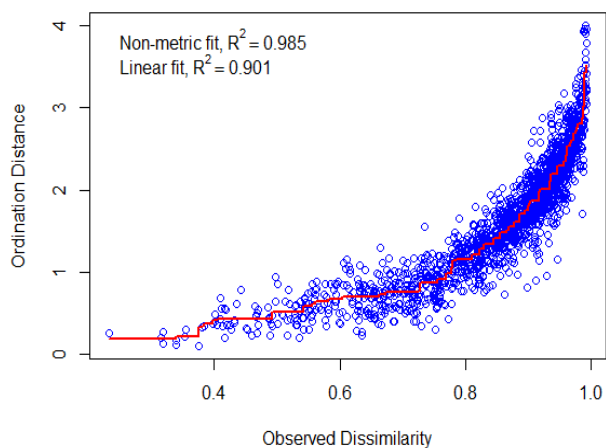

Supplement: Supplementary file 4 — Non-metric multidimensional scaling 2. Non-metric multidimensional scaling ordination of the most abundant bacterial OTUs, stacked bars representing the five most dominant bacterial families and table showing the 5 most abundant genera in adults (Amax), related to A) the mosquito species and B) the feeding status. C) Shepard stress diagram related to the NMDS of Amax sample subset. (PDF 239 kb) [file 40168_2018_528_MOESM4_ESM.pdf]

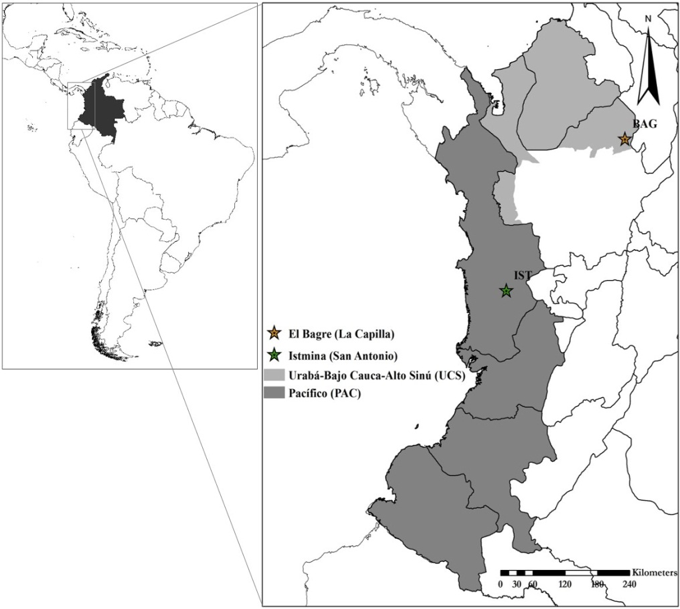

Supplement: Supplementary file 5 — Map. Sampled municipalities belonging to two important epidemiological regions of Colombia: Urabá-Bajo Cauca-Alto Sinú (UCS) and the Pacific (PAC) regions. (PNG 169 kb) [file 40168_2018_528_MOESM5_ESM.png]
