# Supplementary material for: Factors shaping the gut bacterial community assembly in two main Colombian malaria vectors
Source: Microbiome. 2018 Aug 27;6:148. doi: 10.1186/s40168-018-0528-y (PMC6112144; doi:10.1186/s40168-018-0528-y)
Supplement: Supplementary file 6 — Barcoding primers. Detail of barcoding primers used for a directional sequencing. (DOCX 19 kb) [file 40168_2018_528_MOESM6_ESM.docx]

**Additional file 4. Detail of barcoding primers used for directional sequencing**

| **Barcoding Primer** | **Primer** | **Barcode** |
| --- | --- | --- |
| DS01 | CAAGCAGAAGACGGCATACGAGATAACCGCGTGACTGGAGTTC | AACCGC |
| DS02 | CAAGCAGAAGACGGCATACGAGATAACGCCGTGACTGGAGTTC | AACGCC |
| DS03 | CAAGCAGAAGACGGCATACGAGATAAGCGGGTGACTGGAGTTC | AAGCGG |
| DS04 | CAAGCAGAAGACGGCATACGAGATAAGGCGGTGACTGGAGTTC | AAGGCG |
| DS05 | CAAGCAGAAGACGGCATACGAGATACACAGGTGACTGGAGTTC | ACACAG |
| DS06 | CAAGCAGAAGACGGCATACGAGATACACTCGTGACTGGAGTTC | ACACTC |
| DS07 | CAAGCAGAAGACGGCATACGAGATACAGACGTGACTGGAGTTC | ACAGAC |
| DS08 | CAAGCAGAAGACGGCATACGAGATACAGTGGTGACTGGAGTTC | ACAGTG |
| DS09 | CAAGCAGAAGACGGCATACGAGATACCACTGTGACTGGAGTTC | ACCACT |
| DS10 | CAAGCAGAAGACGGCATACGAGATACCAGAGTGACTGGAGTTC | ACCAGA |
| DS11 | CAAGCAGAAGACGGCATACGAGATACCTCAGTGACTGGAGTTC | ACCTCA |
| DS12 | CAAGCAGAAGACGGCATACGAGATACCTGTGTGACTGGAGTTC | ACCTGT |
| DS13 | CAAGCAGAAGACGGCATACGAGATACGACAGTGACTGGAGTTC | ACGACA |
| DS14 | CAAGCAGAAGACGGCATACGAGATACGAGTGTGACTGGAGTTC | ACGAGT |
| DS15 | CAAGCAGAAGACGGCATACGAGATACGTCTGTGACTGGAGTTC | ACGTCT |
| DS16 | CAAGCAGAAGACGGCATACGAGATACGTGAGTGACTGGAGTTC | ACGTGA |
| DS17 | CAAGCAGAAGACGGCATACGAGATACTCACGTGACTGGAGTTC | ACTCAC |
| DS18 | CAAGCAGAAGACGGCATACGAGATACTCTGGTGACTGGAGTTC | ACTCTG |
| DS19 | CAAGCAGAAGACGGCATACGAGATACTGAGGTGACTGGAGTTC | ACTGAG |
| DS20 | CAAGCAGAAGACGGCATACGAGATACTGTCGTGACTGGAGTTC | ACTGTC |
| DS21 | CAAGCAGAAGACGGCATACGAGATAGACACGTGACTGGAGTTC | AGACAC |
| DS22 | CAAGCAGAAGACGGCATACGAGATAGACTGGTGACTGGAGTTC | AGACTG |
| DS23 | CAAGCAGAAGACGGCATACGAGATAGAGAGGTGACTGGAGTTC | AGAGAG |
| DS24 | CAAGCAGAAGACGGCATACGAGATAGAGTCGTGACTGGAGTTC | AGAGTC |
| DS25 | CAAGCAGAAGACGGCATACGAGATAGCACAGTGACTGGAGTTC | AGCACA |
| DS26 | CAAGCAGAAGACGGCATACGAGATAGCAGTGTGACTGGAGTTC | AGCAGT |
| DS27 | CAAGCAGAAGACGGCATACGAGATAGCTCTGTGACTGGAGTTC | AGCTCT |
| DS28 | CAAGCAGAAGACGGCATACGAGATAGCTGAGTGACTGGAGTTC | AGCTGA |
| DS29 | CAAGCAGAAGACGGCATACGAGATAGGACTGTGACTGGAGTTC | AGGACT |
| DS30 | CAAGCAGAAGACGGCATACGAGATAGGAGAGTGACTGGAGTTC | AGGAGA |
| DS31 | CAAGCAGAAGACGGCATACGAGATAGGTCAGTGACTGGAGTTC | AGGTCA |
| DS32 | CAAGCAGAAGACGGCATACGAGATAGGTGTGTGACTGGAGTTC | AGGTGT |
| DS33 | CAAGCAGAAGACGGCATACGAGATAGTCAGGTGACTGGAGTTC | AGTCAG |
| DS34 | CAAGCAGAAGACGGCATACGAGATAGTCTCGTGACTGGAGTTC | AGTCTC |
| DS35 | CAAGCAGAAGACGGCATACGAGATAGTGACGTGACTGGAGTTC | AGTGAC |
| DS36 | CAAGCAGAAGACGGCATACGAGATAGTGTGGTGACTGGAGTTC | AGTGTG |
| DS37 | CAAGCAGAAGACGGCATACGAGATATCCGGGTGACTGGAGTTC | ATCCGG |
| DS38 | CAAGCAGAAGACGGCATACGAGATATCGCGGTGACTGGAGTTC | ATCGCG |
| DS39 | CAAGCAGAAGACGGCATACGAGATATCGGCGTGACTGGAGTTC | ATCGGC |
| DS40 | CAAGCAGAAGACGGCATACGAGATATGCCGGTGACTGGAGTTC | ATGCCG |
| DS41 | CAAGCAGAAGACGGCATACGAGATATGCGCGTGACTGGAGTTC | ATGCGC |
| DS42 | CAAGCAGAAGACGGCATACGAGATATGGCCGTGACTGGAGTTC | ATGGCC |
| DS43 | CAAGCAGAAGACGGCATACGAGATCAACCTGTGACTGGAGTTC | CAACCT |
| DS44 | CAAGCAGAAGACGGCATACGAGATCAACGAGTGACTGGAGTTC | CAACGA |
| DS45 | CAAGCAGAAGACGGCATACGAGATCAAGCAGTGACTGGAGTTC | CAAGCA |
| DS46 | CAAGCAGAAGACGGCATACGAGATCAAGGTGTGACTGGAGTTC | CAAGGT |
| DS47 | CAAGCAGAAGACGGCATACGAGATCACAAGGTGACTGGAGTTC | CACAAG |
| DS48 | CAAGCAGAAGACGGCATACGAGATCACATCGTGACTGGAGTTC | CACATC |
| DS49 | CAAGCAGAAGACGGCATACGAGATCACTACGTGACTGGAGTTC | CACTAC |
| DS50 | CAAGCAGAAGACGGCATACGAGATCACTTGGTGACTGGAGTTC | CACTTG |
| DS51 | CAAGCAGAAGACGGCATACGAGATCAGAACGTGACTGGAGTTC | CAGAAC |
| DS52 | CAAGCAGAAGACGGCATACGAGATCAGATGGTGACTGGAGTTC | CAGATG |
| DS53 | CAAGCAGAAGACGGCATACGAGATCAGTAGGTGACTGGAGTTC | CAGTAG |
| DS54 | CAAGCAGAAGACGGCATACGAGATCAGTTCGTGACTGGAGTTC | CAGTTC |
| DS55 | CAAGCAGAAGACGGCATACGAGATCATCCAGTGACTGGAGTTC | CATCCA |
| DS56 | CAAGCAGAAGACGGCATACGAGATCATCGTGTGACTGGAGTTC | CATCGT |
| DS57 | CAAGCAGAAGACGGCATACGAGATCATGCTGTGACTGGAGTTC | CATGCT |
| DS58 | CAAGCAGAAGACGGCATACGAGATCATGGAGTGACTGGAGTTC | CATGGA |
| DS59 | CAAGCAGAAGACGGCATACGAGATCCAACGGTGACTGGAGTTC | CCAACG |
| DS60 | CAAGCAGAAGACGGCATACGAGATCCAAGCGTGACTGGAGTTC | CCAAGC |
| DS61 | CAAGCAGAAGACGGCATACGAGATCCATCCGTGACTGGAGTTC | CCATCC |
| DS62 | CAAGCAGAAGACGGCATACGAGATCCATGGGTGACTGGAGTTC | CCATGG |
| DS63 | CAAGCAGAAGACGGCATACGAGATCCGCAAGTGACTGGAGTTC | CCGCAA |
| DS64 | CAAGCAGAAGACGGCATACGAGATCCGCTTGTGACTGGAGTTC | CCGCTT |
| DS65 | CAAGCAGAAGACGGCATACGAGATCCGGATGTGACTGGAGTTC | CCGGAT |
| DS66 | CAAGCAGAAGACGGCATACGAGATCCGGTAGTGACTGGAGTTC | CCGGTA |
| DS67 | CAAGCAGAAGACGGCATACGAGATCCTACCGTGACTGGAGTTC | CCTACC |
| DS68 | CAAGCAGAAGACGGCATACGAGATCCTAGGGTGACTGGAGTTC | CCTAGG |
| DS69 | CAAGCAGAAGACGGCATACGAGATCCTTCGGTGACTGGAGTTC | CCTTCG |
| DS70 | CAAGCAGAAGACGGCATACGAGATCCTTGCGTGACTGGAGTTC | CCTTGC |
| DS71 | CAAGCAGAAGACGGCATACGAGATCGAACCGTGACTGGAGTTC | CGAACC |
| DS72 | CAAGCAGAAGACGGCATACGAGATCGAAGGGTGACTGGAGTTC | CGAAGG |
| DS73 | CAAGCAGAAGACGGCATACGAGATCGATCGGTGACTGGAGTTC | CGATCG |
| DS74 | CAAGCAGAAGACGGCATACGAGATCGATGCGTGACTGGAGTTC | CGATGC |
| DS75 | CAAGCAGAAGACGGCATACGAGATCGCCAAGTGACTGGAGTTC | CGCCAA |
| DS76 | CAAGCAGAAGACGGCATACGAGATCGCCTTGTGACTGGAGTTC | CGCCTT |
| DS77 | CAAGCAGAAGACGGCATACGAGATCGCGATGTGACTGGAGTTC | CGCGAT |
| DS78 | CAAGCAGAAGACGGCATACGAGATCGCGTAGTGACTGGAGTTC | CGCGTA |
| DS79 | CAAGCAGAAGACGGCATACGAGATCGGCATGTGACTGGAGTTC | CGGCAT |
| DS80 | CAAGCAGAAGACGGCATACGAGATCGGCTAGTGACTGGAGTTC | CGGCTA |
| DS81 | CAAGCAGAAGACGGCATACGAGATCGTACGGTGACTGGAGTTC | CGTACG |
| DS82 | CAAGCAGAAGACGGCATACGAGATCGTAGCGTGACTGGAGTTC | CGTAGC |
| DS83 | CAAGCAGAAGACGGCATACGAGATCGTTCCGTGACTGGAGTTC | CGTTCC |
| DS84 | CAAGCAGAAGACGGCATACGAGATCGTTGGGTGACTGGAGTTC | CGTTGG |
| DS85 | CAAGCAGAAGACGGCATACGAGATCTACCAGTGACTGGAGTTC | CTACCA |
| DS86 | CAAGCAGAAGACGGCATACGAGATCTACGTGTGACTGGAGTTC | CTACGT |
| Barcode primer R | AATGATACGGCGACCACCGAGATCTACACTCTTTCCCTACACGAC |  |
