# Supplementary material for: Factors shaping the gut bacterial community assembly in two main Colombian malaria vectors
Source: Microbiome. 2018 Aug 27;6:148. doi: 10.1186/s40168-018-0528-y (PMC6112144; doi:10.1186/s40168-018-0528-y)
Supplement: Supplementary file 7 — Rarefied vs non-rarefied data. Non-metric multidimensional scaling ordinations, stacked bar plots and tables are shown as examples of the small variations observed after an exploratory analysis of the data comparing rarefied versus non-rarefied data. (PDF 241 kb) [file 40168_2018_528_MOESM7_ESM.pdf]

Rarefied

Not rarefied

Fig 1A

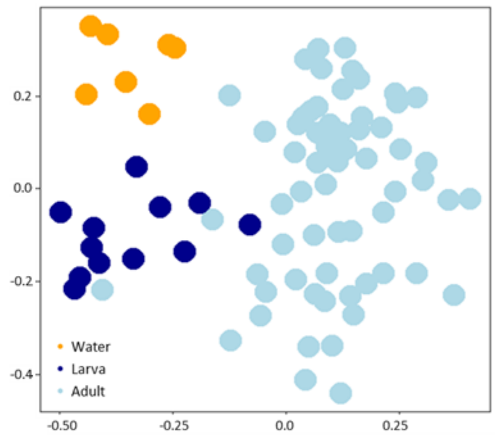

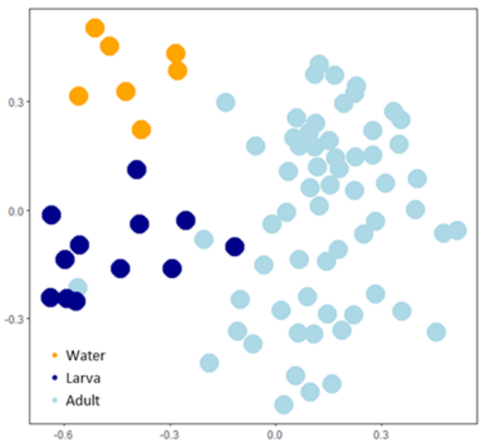

Fig 2C

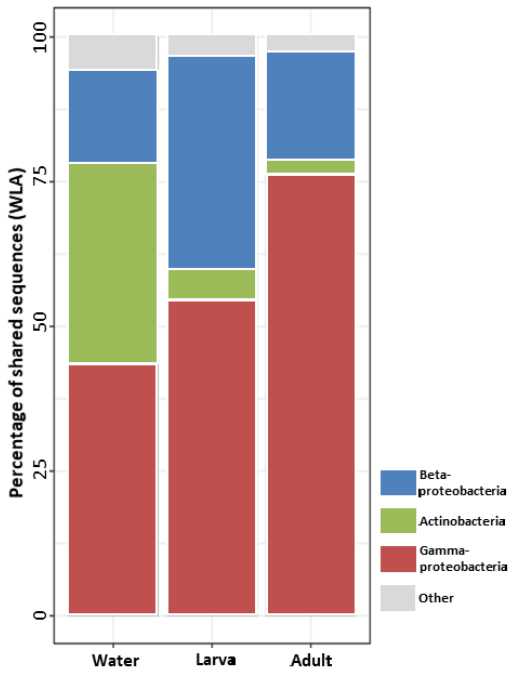

Rarefied
N=1532

Class	Ws	Larva	Adult
Gammaproteobacteria	43.57126	54.49485	76.2576
Actinobacteria	34.63802	5.518846	2.325185
Betaproteobacteria	15.72042	37.41617	19.2701

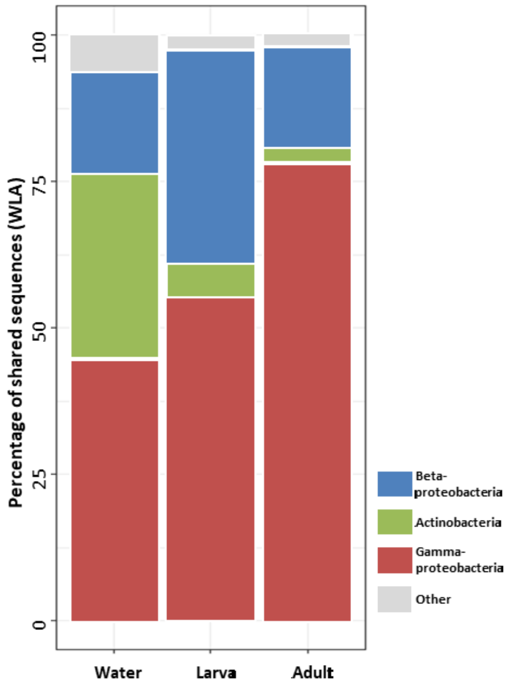

Not Rarefied
N=2142

Class	Ws	Larva	Adults
Gammaproteobacteria	44.18522	54.88268	78.37768
Actinobacteria	31.77652	6.03035	2.382757
Betaproteobacteria	18.05361	36.45133	17.05406

Additional file 3A

Genus	An. nuneztovari	An. darlingi
NA	45.11	40.84
Pseudomonas	19.34	17.97
Acinetobacter	5.17	13.61
Leptothrix	11.67	5.82
Aeromonas	1.31	5.02
Thorsellia	5.67	4.40

Genus	An. nuneztovari	An. darlingi
NA	49.00	42.06
Pseudomonas	20.24	17.97
Acinetobacter	7.89	12.34
Leptothrix	9.76	5.17
Aeromonas	1.20	5.31
Thorsellia	3.92	3.97
